# Riboflavin overproduction on lignocellulose hydrolysate by the engineered yeast *Candida famata*

**DOI:** 10.1093/femsyr/foae020

**Published:** 2024-07-15

**Authors:** Ljubov S Dzanaeva, Dominik Wojdyła, Dariya V Fedorovych, Justyna Ruchala, Kostyantyn V Dmytruk, Andriy A Sibirny

**Affiliations:** Institute of Cell Biology, National Academy of Sciences of Ukraine, Drahomanov St., 14/16, 79005 Lviv, Ukraine; Institute of Biotechnology, University of Rzeszow, Zelwerowicza 4, 35-601 Rzeszow, Poland; Institute of Cell Biology, National Academy of Sciences of Ukraine, Drahomanov St., 14/16, 79005 Lviv, Ukraine; Institute of Biotechnology, University of Rzeszow, Zelwerowicza 4, 35-601 Rzeszow, Poland; Institute of Cell Biology, National Academy of Sciences of Ukraine, Drahomanov St., 14/16, 79005 Lviv, Ukraine; Institute of Cell Biology, National Academy of Sciences of Ukraine, Drahomanov St., 14/16, 79005 Lviv, Ukraine; Institute of Biotechnology, University of Rzeszow, Zelwerowicza 4, 35-601 Rzeszow, Poland

**Keywords:** riboflavin, *Candida famata*, yeast, riboflavin overproducers, lignocellulose hydrolysate, xylose

## Abstract

Lignocellulose (dry plant biomass) is an abundant cheap inedible residue of agriculture and wood industry with great potential as a feedstock for biotechnological processes. Lignocellulosic substrates can serve as valuable resources in fermentation processes, allowing the production of a wide array of chemicals, fuels, and food additives. The main obstacle for cost-effective conversion of lignocellulosic hydrolysates to target products is poor metabolism of the major pentoses, xylose and L-arabinose, which are the second and third most abundant sugars of lignocellulose after glucose. We study the oversynthesis of riboflavin in the flavinogenic yeast *Candida famata* and found that all major lignocellulosic sugars, including xylose and L-arabinose, support robust growth and riboflavin synthesis in the available strains of *C. famata*. To further increase riboflavin production from xylose and lignocellulose hydrolysate, genes *XYL1* and *XYL2* coding for xylose reductase and xylitol dehydrogenase were overexpressed. The resulting strains exhibited increased riboflavin production in both shake flasks and bioreactors using diluted hydrolysate, reaching 1.5 g L^−1^.

## Teaser

The current manuscript represents the first publication on riboflavin oversynthesis using lignocellulosic hydrolysates.

## Take-away

- Lignocellulosic hydrolysates support vigorous growth of the yeast *C. famata*.
- Available strains of *C. famata* overproduce riboflavin on hydrolysates.
- Overexpression of *XYL1* gene improved riboflavin production.

## Introduction

Riboflavin (vitamin B_2_) is a water-soluble vitamin, produced by all plants and most of microorganisms. It is essential for growth and reproduction of humans and animals. Riboflavin is the precursor of flavin nucleotides (FMN and FAD), crucial coenzymes involved in various oxidoreductive reactions (Abbas and Sibirny 2011, Schwechheimer et al. 2016). Riboflavin is an important biotechnological product that is used mainly in agriculture as a feed additive, as well as in the food industry and medicine (Beztsinna et al. 2016, You et al. 2021). Riboflavin is currently biotechnologically produced by engineered strains of the bacterium *Bacillus subtilis* and the filamentous fungus, *Ashbya (Eremothecium) gossypii* (Ruchala et al. 2022). To enhance microbial riboflavin production, strains were improved through metabolic engineering and classical selection (Zhao et al. 2021). These approaches involved random mutagenesis induced by chemical exposure and UV irradiation, as well as random and site-directed mutagenesis achieved through genetically engineered deletions, insertions, or substitutions (Zhao et al. 2021). Additionally, the fermentation process was optimized by selecting and adjusting medium components and their concentrations (You et al. 2021).

However, further research is required to enhance industrial riboflavin processes, focusing on improving key steps, including fermentation conditions, purification techniques, and the utilization of recycled sources. The price of riboflavin depends significantly on the price of the carbon substrate used (Kato and Park 2012). It would be especially important to use waste as a carbon source.

In our current work, we studied riboflavin production by the yeast *Candida famata* (teleomorph form is known as *Debaryomyces subglobosus* Nguyen et al. 2009). The riboflavin overproducing strain of *C. famata* was used for more than 10 years for industrial riboflavin production. However, its production was discontinued due to genetic instability of the used strain (Abbas and Sibirny 2011). We constructed stable non-reverting riboflavin-overproducing strains of *C. famata* using a combination of classical selection methods and metabolic engineering. These strains accumulated 1–1.45 g L^−1^ of riboflavin in flasks and reached 16.4 g L^−1^ in a 7 L bioreactor during fed-batch cultivation (Dmytruk et al. 2011, Dmytruk et al. 2014). A yeast strain overexpressing the *RFE1* gene, which encodes riboflavin excretase, produced 1.7 g L^−1^ of vitamin B_2_ (Tsyrulnyk et al. 2020). Modulation of the purine biosynthesis pathway further enhanced riboflavin production, resulting in up to 2.85 g L^−1^ (Dmytruk et al. 2020). The engineered strains produced riboflavin in media with glucose as the carbon source. The latest strain, expressing Sef1—a positive regulator of riboflavin synthesis—under a lactose-inducible promoter, or the structural *RIB6* gene, achieved riboflavin production of approximately 2.5 g L^−1^ using milk whey, a byproduct of cheese production, as the carbon source (Ruchala et al. 2022).

Lignocellulosic hydrolysates can serve as valuable resources for fermentation processes, allowing the production of a wide range of chemicals, fuels, and food additives. For instance, ethanol (Robak and Balcerek 2020), organic acids (Jimenez-Quero et al. 2020), polymers (Kawaguchi et al. 2017), and enzymes (Namnuch et al. 2021) are among the diverse products that can be synthesized through this approach. Different yeast species have been considered for the production of chemicals, mainly ethanol and xylitol, using different lignocellulosic substrates, such as horticultural waste olive tree pruning, rice straw, corncob, and sugarcane bagasse (Bergmann et al. 2019).

In this study, we demonstrated that engineered strains of *C. famata* exhibit riboflavin overproduction in medium containing lignocellulose hydrolysate. We investigated both, previously isolated, and newly constructed recombinant strains with overexpression of *XYL1* and *XYL2* genes encoding for xylose reductase and xylitol dehydrogenase, respectively, for their riboflavin production capabilities in flask and bioreactor experiments.

## Materials and methods

### Strains, media, and cultivation conditions

*C. famata* VKMY-9 (wild type) and BRP/PRS3m/ADE4 m (designated as BRPI from the Best Riboflavin Producer Improved) (Dmytruk et al. 2020) strains were used in this study. Riboflavin oversynthesis by BRPI is independent of iron ions. Yeast strains were cultivated at 30°C on rich YPD (5 g L^−1^ yeast extract, 10 g L^−1^ peptone, and 20 g L^−1^ glucose) or mineral YNB (1.7 g L^−1^ yeast nitrogen base, 5 g L^−1^ ammonium sulphate and 20 g L^−1^ glucose or other carbon sources) media. The modified YNB medium (containing 1.7 g L^−1^ yeast nitrogen base, 3 g L^−1^ ammonium sulfate, and 2 g L^−1^ yeast extract) was supplemented with three, four, or five times diluted hydrolysate for cultivation in flasks or bioreactors. Sugarcane straw (bagasse) hydrolysate was prepared using liquid hot water pretreatment before enzymatic hydrolysis, following the method described by Zhuang et al. 2016 and Jimenez-Gutierrez et al. 2021 and supplied by a GranBio Investimentos S.A. (Brazil). The hydrolysate, with a pH of 5, which contained glucose (63.35 g L^−1^), xylose (34.48 g L^−1^), galactose (1.20 g L^−1^), L-arabinose (3.43 g L^−1^), mannose (1.22 g L^−1^), acetic acid (5.22 g L^−1^), formic acid (0.93 g L^−1^), furfural (0.62 g L^−1^), hydroxymethylfurfural (HMF) (0.43 g L^−1^) (Table S1), was added to the medium at various dilution rates.

*Escherichia coli* strain DH5α (*Φ80dlacZ∆M15, recA1, endA1, gyrA96, thi-1, hsdR17(r-K m + K), supE44, relA1, deoR, ∆(lacZYA-argF) U169*) was used in experiments that required a bacterial host. DH5α was grown at 37°C in LB medium as described (Sambrook et al. 1989). Transformed *E. coli* cells were maintained in rich medium containing 100 mg L^−1^ of ampicillin.

To estimate riboflavin synthesis the yeast cells from a fresh plate were grown in 50 mL of liquid media in 250 mL Erlenmeyer flasks with initial biomass 5 mg L^−1^. To prepare for bioreactor cultivation, a preculture of *C. famata* strains was incubated with shaking at 220 rpm and 28°C for 24 h in YPD. This preculture was used to inoculate the bioreactors with a starting volume of 500 mL of batch medium and a starting biomass of 1 g CDW L^−1^. Fermentations were carried out in 1 Liter total volume bioreactors (Sartorius Biostat® B2). The fermentation temperature was maintained at 28°C, the pH was controlled at 5.5 ± 0.5 by adding 100 g L^−1^ sodium hydroxide, and the dissolved oxygen concentration was maintained at 70% saturation by controlling the agitation speed between 200 and 500 rpm, while the airflow was kept constant at 1.5 L min^−1^.

### Plasmids and strains constructions

Standard cloning techniques were used as described (Sambrook et al. 1989). Genomic DNA of *C. famata* was isolated using the NucleoSpin® Tissue Kit (Macherey-Nagel, Duren, Germany). Restriction endonucleases and DNA ligase (Thermo Fisher Scientific Baltics, Vilnius, Lithuania) were used following the manufacturer's specifications. Plasmid isolation from *E. coli* was performed with the ZyppyTM Plasmid Miniprep (Irvine, CA, USA). PCR amplification of the fragments of interest was performed using Phusion High-Fidelity DNA Polymerase (Thermo Fisher Scientific Baltics, Vilnius, Lithuania) according to the manufacturer's specification. PCRs were performed in a GeneAmp PCR System 9700 thermocycler (Applied Biosystems, Foster City, CA, USA).

The nourzeotricin resistance gene *SAT-1* was amplified from the pTb/SAT-1/R1 (Petrovska et al. 2022) using the primer pair Ko1258 (CGG GGT ACC AGT CTT ATA TAT ATC CGA ACT TGG)/Ko1259 (CCG GAA TTC TCA CAT AAC CAC AAG GTG CC). The KpnI and EcoRI restriction sites were incorporated into the Ko1258 and Ko1259 respectively. The *SAT-1* was treated with KpnI and EcoRI, and directly cloned into the plasmid pUC57_prTEF1*Cf*_trTEF1*Dh*_Ble_Sa (Ruchala et al. 2022) digested with KpnI and EcoRI, instead of Ble_Sa. The resulting plasmid was designated as pT-SAT.

**Figure 1.**
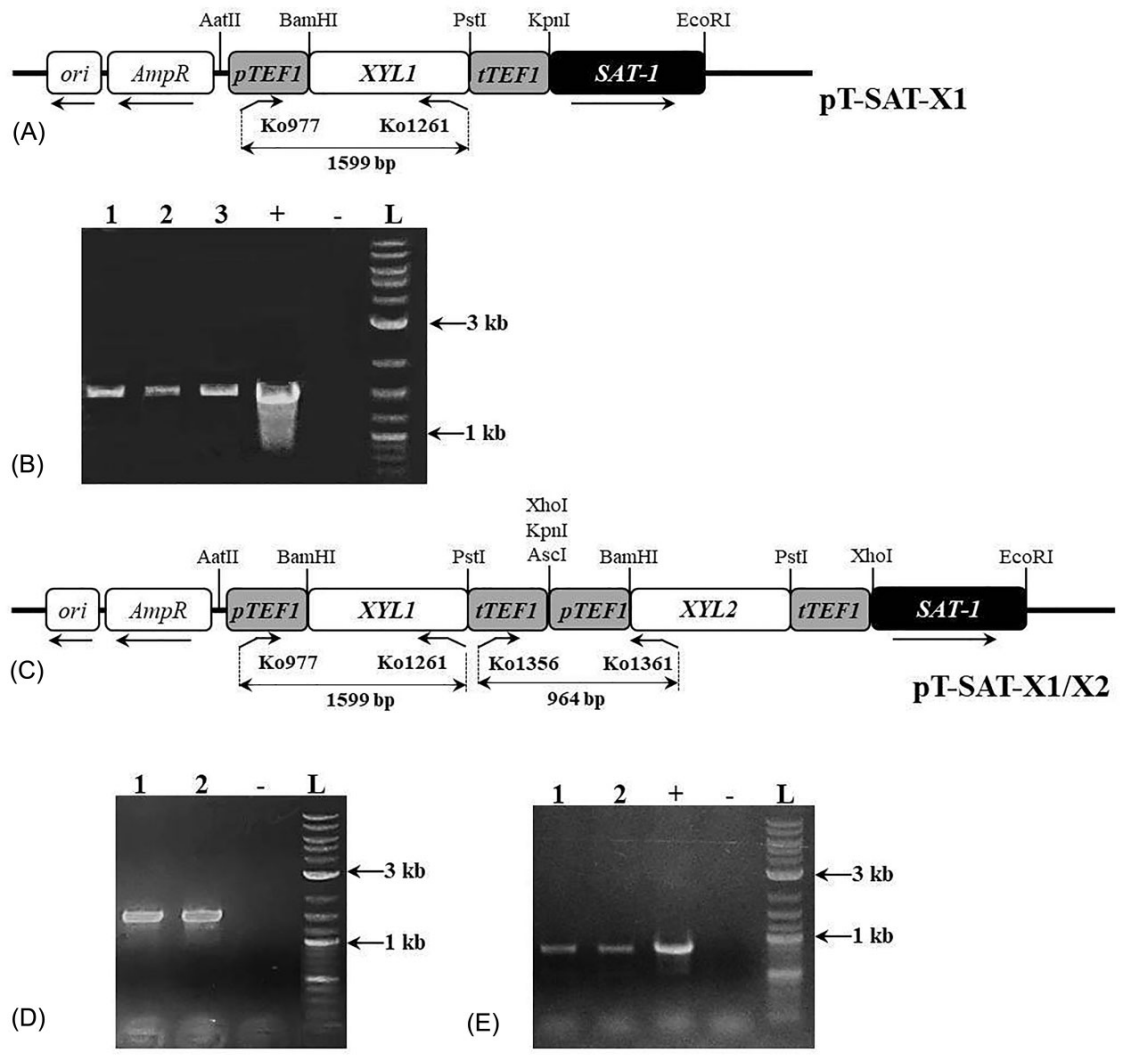
(A, C) Linear scheme of plasmids pT-SAT-X1 and pT-SAT-X1/X2. The promoter of TEF1 C. famata and terminator of the TEF1 D. hansenii are indicated as grey boxes; XYL1 and XYL2 C. famata—as open boxes; selective marker SAT-1–as a black box. (B, D) PCR verification of BRPI/XYL1 and BRPI/XYL1/XYL2 strains with pair of primers Ko977/Ko1261, which amplify 1599 bp fragment (positive and negative controls indicated as + and −; 1–3–BRPI/XYL1, 1–2–BRPI/XYL1/XYL2 and L—1 kb, 3 kb, ladder). (E) PCR verification of BRPI/XYL1/XYL2 strains with pair of primers Ko1356/Ko1361, which amplify 964 bp fragment. (positive and negative controls indicated as + and −; 1–2–BRPI/XYL1/XYL2, and L—1 kb, 3 kb, ladder).

The *XYL1* gene was amplified from genomic DNA of VKMY-9 using the pair Ko1260 (CGC GGA TCC ATG TCT ATT AAG TTG AAT TCA GGA TAT G)/Ko1261 (AAA CTG CAG TTA AGC AAA GAT TGG AAT CTT GTC C). The BamHI and PstI restriction sites were incorporated into the Ko1260 and Ko1261 respectively. After the BamHI/PstI restriction the *XYL1* was cloned between *TEF1* promoter and terminator into the corresponding sites of pT-SAT to create pT-SAT-X1 (Fig. 1A).

The *XYL2* gene was amplified from genomic DNA of VKMY-9 using a pair of primers Ko1359 (CGC GGA TCC ATG ACA CCA AAC CCT TCC TTA G)/Ko1360 (AAA CTG CAG TTA TTC TGG ACC ACT GAT GAT AC). The BamHI and PstI restriction sites were incorporated into primers Ko1359 and Ko1360 respectively. After the BamHI/PstI restriction the *XYL2* was cloned between *TEF1* promoter and terminator into the corresponding sites of pT-SAT to create pT-SAT-X2. The *XYL2* gene with *TEF1* promoter and terminator was amplified using the pair Ko1362 (CCG CTC GAG AAA TTG ACT GGT CTG AAA TAA TAG)/Ko1363 (CCG CTC GAG ATG TTG CGC CGA ACA ATC AC), treated with XhoI and cloned into XhoI-linearized pT-SAT-X1 to create pT-SAT-X1/X2 (Fig. 1C).

Plasmids pT-SAT-X1 and pT-SAT-X1/X2 were linearized with the restriction endonuclease AatII and used for transformation of the BRPI. The transformants were selected on a solid mineral medium, containing nourseothricin at 20 mg L^−1^ on the third day of incubation. Subsequently, the selected strains BRPI/XYL1 and BRPI/XYL1/XYL2 were checked by PCR using a pair of primers Ko977 (CCC AAG CTT AAA TTG ACT GGT CTG AAA TAA TAG)/Ko1261 to verify the presence of the expression module of *XYL1* (Fig. 1B, D) and Ko1356/Ko1361 to verify of presence the expression module of *XYL2* (Fig. 1E). The selected transformants were stabilized through cultivation in non-selective media for 12–14 generations, followed by a shift to selective media using replica plating. The same round of stabilization was repeated after the storage of the selected strains at -80°C for three months. The presence of corresponding expression modules in the stabilized transformants was confirmed via PCR using the same primers.

### Quantitative Real-Time PCR

The expression of the *XYL1* gene was confirmed by qRT-PCR. Total RNA was extracted from yeast cells using the GeneMATRIX Universal RNA Purification Kit with DNAseI (EURx Ltd., Gdansk, Poland). The qRT-PCR was performed by 7500 Fast Real-Time PCR System (The Applied Biosystems, USA) with a SG OneStep qRT-PCR kit (EURx Ltd., Gdansk, Poland) using corresponding pairs of primers XYL1_Cf_f_qRT (ATG TCT ATT AAG TTG AAT TCA GGA TAT)/XYL1_Cf_r_qRT (TTA AGC AAA GAT TGG AAT CTT GTC), XYL2_f_Cf (GTG GAC GCC ATA TTC AAA TTG)/XYL2_r_Cf (GTC ATA AGC GTC AAT AGC TTC), and ACT1f (TAA GTG TGA TGT CGA TGT CAG)/ACT1r (TTT GAG ATC CAC ATT TGT TGG AA); RNA as a template; and ROX reference passive dye following the manufacturer's instructions as previously described (Dmytruk et al. 2020).

### Biochemical analysis

The cell biomass of yeast was determined with a Helios Gamma spectrophotometer (OD, 590 nm; cuvette, 10 mm) turbidimetrically with gravimetric calibration. Flavin production was analyzed by measuring fluorescence (Turner Quantech FM 109510–33 fluorometer, excitation maximum at 440 nm, emission maximum at 535 nm).

Concentrations of glucose and xylose in the medium broth were analyzed by HPLC (PerkinElmer, Series 2000, USA) with an Aminex HPX-87H ion-exchange column (BioRad, Hercules, USA) and Refractive Index Detector (PerkinElmer, Series 200a, USA). A mobile phase of 4 mM H_2_SO_4_ was used at a flow rate 0.6 mL min^−1^ and the column temperature was 30°C.

### Statistical analysis

All the experimental data shown in this manuscript were collected from at least three independent experiments to ensure reproducibility of the trends and relationships observed in the cultures. A statistical T-test was used. Each error bar indicates the standard deviation (SD) from the mean obtained from the samples in biological triplicate. The 5% significance level was used in the statistical analyses.

## Results

### Riboflavin production by *C. famata* BRPI strain cultivated in media with different carbon sources

A preliminary screening of carbon sources potentially present in lignocellulosic hydrolysates supporting the growth and riboflavin production by *C. famata* was conducted. It was observed that BRPI accumulated biomass on hexoses (glucose, fructose, mannose, and galactose) in the range of 2.9 to 3.3 g L^−1^, as well as on pentoses (xylose and L-arabinose) at 1.9 and 2.6 g L^−1^, respectively (Table 1). Biomass accumulation on pentoses was approximately 25% lower compared to that on hexoses. All tested carbon sources supported riboflavin production by BRPI. Riboflavin production and riboflavin production calculated per cell dry weight (CDW) on glucose or galactose were similar, reaching 812.5 or 757.5 mg L^−1^ and 248.5 or 265.8 mg g CDW^−1^, respectively (Table 1). Despite riboflavin production on xylose was only 337.5 mg L^−1^ the riboflavin production per CDW on this substrate was almost the same as on mannose reaching 170.4 mg g CDW^−1^ (Table 1). Although riboflavin production on xylose was only 337.5 mg L^−1^, the riboflavin production per CDW on this substrate was nearly the same as that on mannose, reaching 170.4 mg g CDW^−1^. Riboflavin production on L-arabinose was 1.46 times higher than that on xylose, reaching 492.5 mg L^−1^ (Table 1).

**Table 1.** Growth and riboflavin production of the *C. famata* BRPI strain in YNB medium supplemented with different sources of carbon: Cultivation time–120 hours.

Carbon sources	Biomass (g L^−1^)	Riboflavin (mg L^−1^)	Riboflavin production per CDW (mg g CDW^−1^)
Glucose	3.3 ± 0.1	812.5 ± 32.5	248.5 ± 12.4
Fructose	3.4 ± 0.1	542.5 ± 24.4	158.8 ± 7.9
Mannose	3.0 ± 0.1	508.2 ± 22.9	171.2 ± 8.6
Galactose	2.9 ± 0.1	757.5 ± 34.1	265.8 ± 13.3
L-arabinose	2.6 ± 0.1	492.5 ± 22.2	190.1 ± 9.5
Xylose	1.9 ± 0.1	337.5 ± 13.5	170.4 ± 8.5

**Figure 2.**
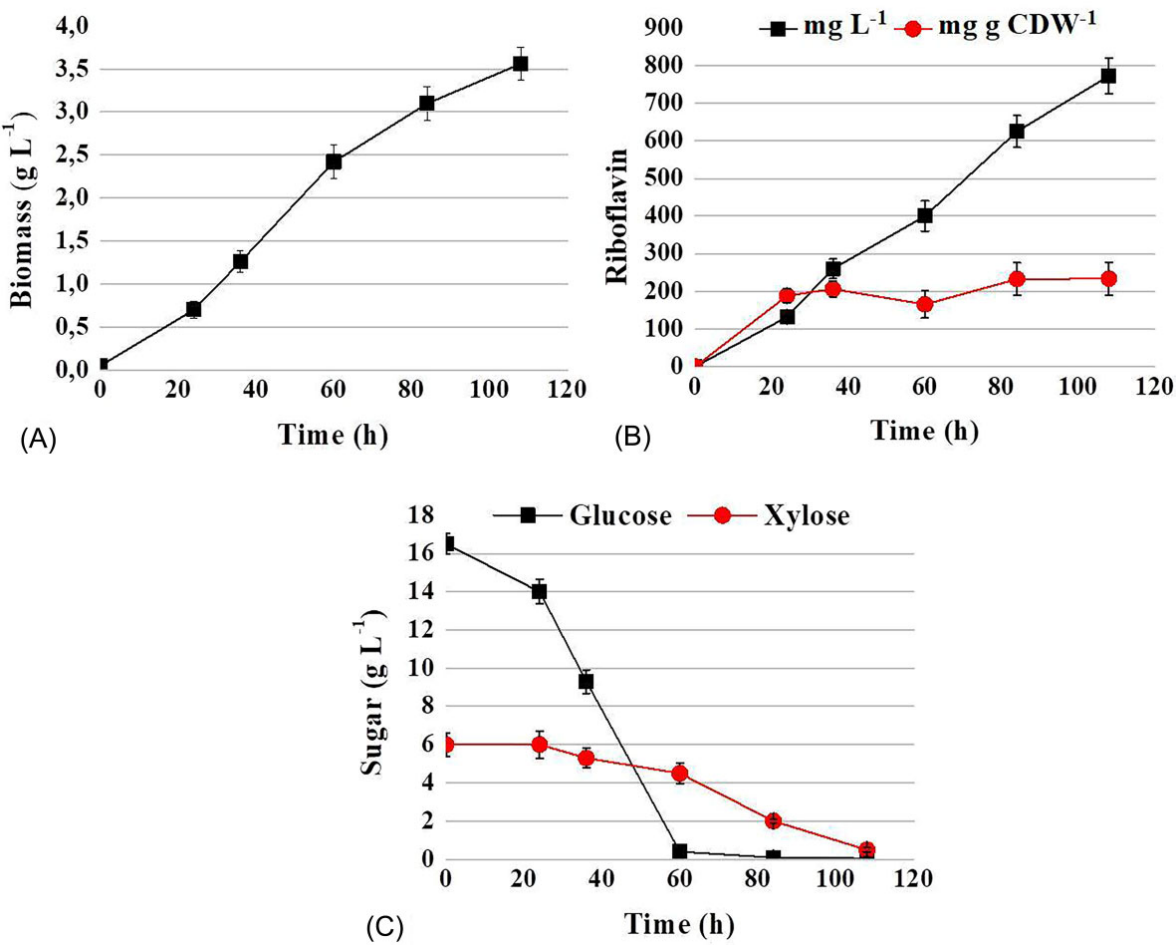
Time profiles of growth (A), riboflavin production and riboflavin production per CDW (B) and sugars consumption (C) in flasks by strain BRPI C. famata on YNB supplemented with glucose and xylose in a ratio of 2.7 to 1 (16 g of glucose and 6 g of xylose).

Since the main components of the lignocellulose hydrolysate are glucose and xylose (see “Material and methods”), we analyzed the riboflavin production of BRPI in a YNB medium containing these sugars in 2.7:1 ratio (16 g of glucose and 6 g of xylose) (Fig. 2).

The strain studied achieved a biomass accumulation of up to 3.6 g L^−1^ in this medium. Riboflavin production reached 772.5 g L^−1^, with a riboflavin production per CDW of 234 mg g CDW^−1^ after 108 hours of cultivation. The yeast consumed glucose within 60 hours of cultivation and xylose within 108 hours.

### Riboflavin production by strain BRPI *C. famata*, grown on media with lignocellulose hydrolysate

Biomass accumulation and riboflavin production by the *C. famata* strain BRPI were assessed in media containing hydrolysate. BRPI was unable to grow in undiluted hydrolysate, likely due to the presence of toxic concentrations of furfural, HMF and acetic acid. Therefore, a modified medium containing diluted hydrolysate was employed. Synthetic YNB medium supplemented with 3 g L^−1^ ammonium sulfate, 2 g L^−1^ yeast extract, and four times diluted hydrolysate supported BRPI growth.

**Figure 3.**
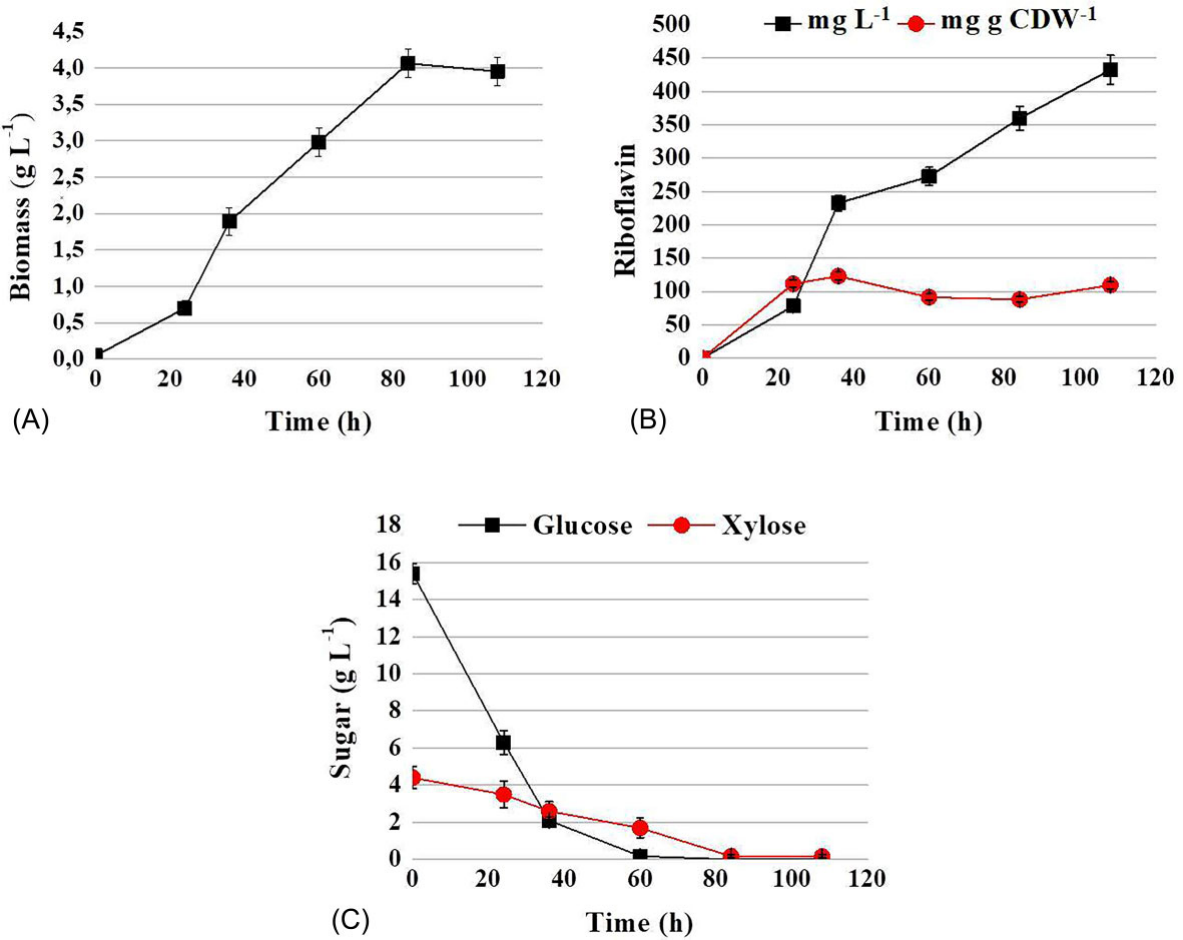
Time profiles of growth (A), riboflavin production and riboflavin production per CDW (B) and sugars consumption (C) in flasks by strain BRPI C. famata using hydrolysate diluted 4 times with synthetic YNB medium supplemented with 2 g L-1 yeast extract.

BRPI strain achieved a biomass accumulation of up to 4.0 g L^−1^ in this medium (Fig. 3). Riboflavin production reached 432.5 g L^−1^, with a riboflavin production per CDW of 109.4 mg g^−1^ of CDW after 108 hours of cultivation (Fig. 3). The yeast consumed completely glucose within 60 hours of cultivation and xylose in 108 hours of cultivation on diluted hydrolysate. It is important to note the simultaneous utilization of glucose and xylose from hydrolysate (Fig. 3).

### Construction of recombinant strains of *C. famata* with expressed *XYL1* and *XYL1/XYL2* genes

As previously observed (Table 1), the production of riboflavin in xylose was relatively low compared to other sugars tested. To enhance xylose conversion to riboflavin, we opted to overexpress the first genes of the xylose utilization pathway, *XYL1* and *XYL2*, which encode xylose reductase and xylitol dehydrogenase, respectively. The BRPI strain of *C. famata* was used as the parental one for overexpression of the *XYL1* and *XYL1/XYL2* genes.

The expression of the *XYL1* gene was analyzed by qRT-PCR in BRPI/XYL1 strain on xylose and glucose as a control. It was found that the expression profiles of *XYL1* on xylose was 3.8-fold increased as compared to that of the parental strain cultivated on xylose with no increase in glucose medium (Table 2). The expression of *XYL1* and *XYL2* in BRPI/XYL1/XYL2 strain on xylose increased by 15 and 15.6-fold, respectively, compared to that of BRPI. An increase in the expression of both these genes in glucose medium was visible, but to a lower extent than in xylose medium (Table 2).

**Table 2.** Cell biomass, riboflavin production and riboflavin yield of the recombinant strains BRPI/XYL1 and BRPI/XYL1/XYL2 of *C. famata* on YNB medium with xylose represented at 96 h of cultivation. Relative expression levels of the *XYL1* and *XYL2* genes in the BRPI/XYL1 and BRPI/XYL1/XYL2 strains versus the BRPI recipient strain. Relative expression levels were obtained using the comparative Ct method for quantification of the ΔΔC_t_ values. The error bars indicate standard deviations calculated from at least two independent experiments performed in triplicates. The strains were grown in YNB medium supplemented with 20 g L^−1^ glucose or 20 g L^−1^ xylose at 30°C, 220 rpm.

Strain	Biomass (g L^−1^)	Riboflavin (mg L^−1^)	Riboflavin production per CDW (mg g CDW^−1^)	Riboflavin yield (mg g^−1^ consumed xylose)	Relative expression of *XYL1* gene		Relative expression of *XYL2* gene	
					glucose	xylose	glucose	xylose
BRPI	2.00 ± 0.12	330 ± 12	165 ± 5	18 ± 0.7	1.00 ± 0.31	1.00 ± 0.29	1.00 ± 0.45	1.00 ± 0.035
BRPI/XYL1	2.13 ± 0.25	450 ± 16	211 ± 6	23 ± 0.7	0.68 ± 0.18	3.82 ± 0.27	0.128 ± 0.06	6.71 ± 0.99
BRPI/XYL1/XYL2	2.25 ± 0.25	474 ± 16	210 ± 6	24 ± 0.7	1.33 ± 0.17	14.98 ± 0.24	2.86 ± 0.042	15.64 ± 1.13

### Growth and riboflavin production by recombinant strains of *C. famata* with overexpressed *XYL1* and *XYL1/XYL2* genes

**Figure 4.**
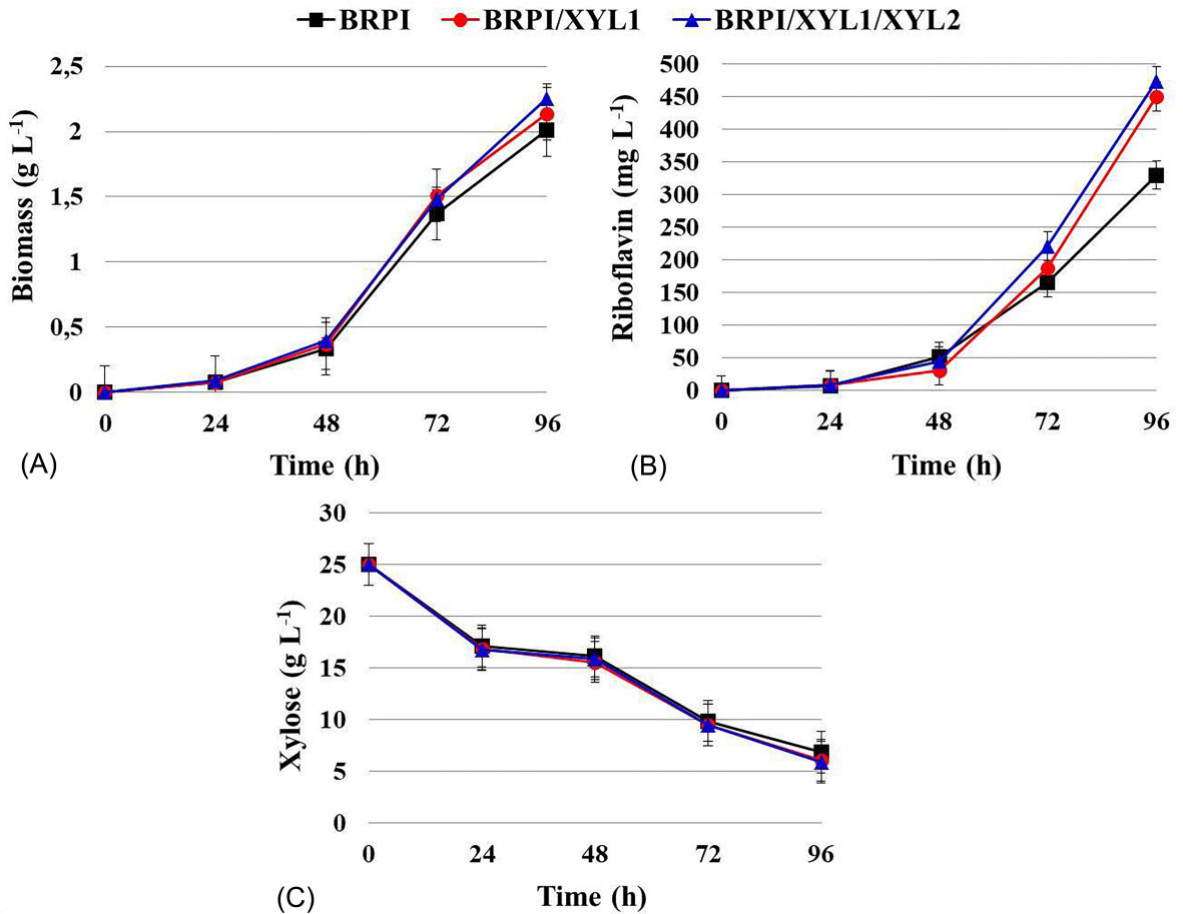
Time courses of biomass accumulation (A), riboflavin production (B), and xylose consumption (C) by C. famata BRPI, BRPI/XYL1 and BRPI/XYL1/XYL2 strains on YNB supplemented with xylose.

Characterization of riboflavin production in the recombinant strains of *C. famata* BRPI/XYL1 and BRPI/XYL1/XYL2 on the YNB medium with xylose was performed after 96 hours of cultivation. The overexpression of *XYL1* and *XYL1/XYL2* led to a slight increase in biomass accumulation, with the respective strains showing a 1.06-fold and 1.13-fold increase compared to BRPI (Fig. 4, Table 2). The strain BRPI/XYL1 produced 450 mg L^−1^ of riboflavin, indicating a 1.36-fold increase of vitamin B2 production compared to that of BRPI (Fig. 4, Table 2). The riboflavin production per CDW and riboflavin yield by BRPI/XYL1 were 211 mg g^−1^ of CDW and 23 mg g^−1^ of xylose, respectively. The strain had a 1.28-fold increase in riboflavin production per g of CDW or riboflavin yield per g of consumed xylose compared to that of the parental BRPI strain (Table 2). Strain BRPI/XYL1/XYL2 demonstrated 1.44-fold increase in riboflavin production that amounted to 474 mg L^−1^ (Fig. 4, Table 2) when compared to the BRPI. The riboflavin production per CDW and riboflavin yield of the BRPI/XYL1/XYL2 amounted to 210 mg g^−1^ of CDW and 24 mg g^−1^ of xylose. The strain had a 1.28-fold or 1.33-fold increase in riboflavin production per g of CDW or riboflavin yield per g of consumed xylose when compared to the parental BRPI strain (Table 2).

**Figure 5.**
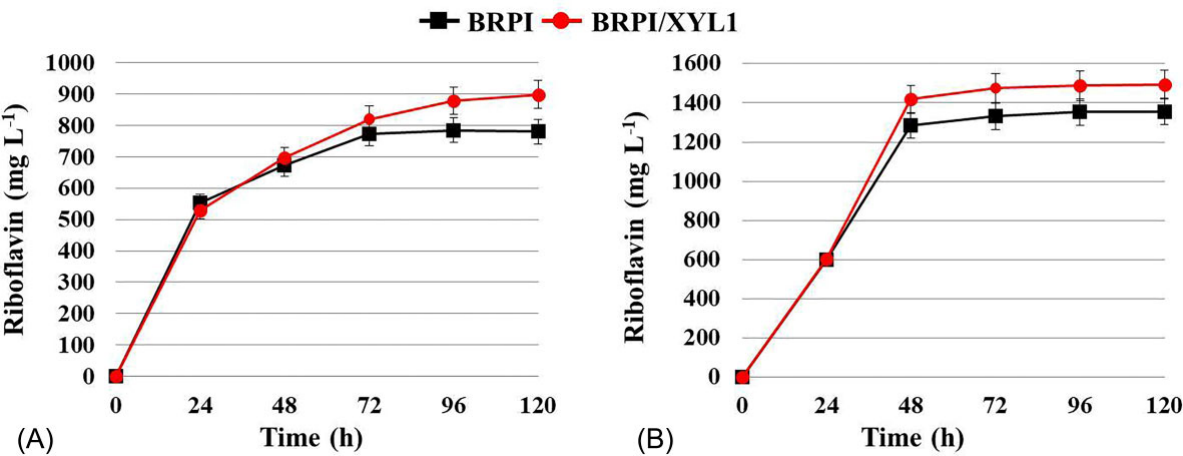
Production of riboflavin by C. famata yeast strains BRPI and BRPI/XYL1 in bioreactors on YNB medium with the addition of five times (A) or three times diluted hydrolysate (B).

The strain BRPI and BRPI/XYL1 were utilized for experiments in the bioreactor. Both were grown in YNB medium supplemented with five or three times diluted bagasse hydrolysate. Over the course of a 5-day bioreactor cultivation using a five times diluted hydrolysate, biomass accumulation and riboflavin production by BRPI and BRPI/XYL1 strains reached 4.9 g L^−1^ and 5.2 g L^−1^, and 780 mg L^−1^ and 900 mg L^−1^, respectively (Fig. 5A; Table 3). The biomass of yeast strains cultivated in a three times diluted hydrolysate was 1.6-fold higher compared to that in a five times diluted hydrolysate, reaching 8.2 g L^−1^ for both strains (Table 3). Riboflavin production by BRPI and BRPI/XYL1 on this medium reached 1356.2 mg L^−1^ and 1493.5 mg L^−1^, respectively (Fig. 5B; Table 3). Hence, the expression of *XYL1* increased riboflavin production and riboflavin yield per consumed glucose and xylose by 15% and 10% in five and three times diluted hydrolysate, respectively (Table 3). BRPI and BRPI/XYL1 completely consumed glucose within 48 hours of cultivation and xylose within 120 hours of cultivation, in a similar manner for both dilution rates. It is noteworthy that biomass accumulation and riboflavin production in three times diluted hydrolysate reached close to their peak values at 48 hours of cultivation, unlike in five times diluted hydrolysate (Fig. 5).

**Table 3.** Cell biomass, riboflavin production, riboflavin production per CDW and riboflavin yield of the recombinant strains BRPI/XYL1 and BRPI/XYL1/XYL2 of *C. famata* under conditions of high cell density cultivation in bioreactor in YNB medium containing five and three times diluted hydrolysate represented at 120 h of cultivation.

Strain	Biomass (g L^−1^)	Riboflavin (mg L^−1^)	Riboflavin production per CDW (mg g CDW^−1^)	Riboflavin yield (mg g^−1^ consumed glucose and xylose)
BRPI^a^	4.9 ± 0.1	780.0 ± 23.1	159.2 ± 4.8	40.3 ± 1.6
BRPI/XYL1^a^	5.2 ± 0.2	900.0 ± 40.3	173.1 ± 6.3	46.5 ± 1.9
BRPI^b^	8.2 ± 0.3	1356.2 ± 56.8	165.4 ± 5.2	41.8 ± 1.8
BRPI/XYL1^b^	8.2 ± 0.3	1493.5 ± 60.4	182.1 ± 8.3	46.1 ± 2.3

a medium containing five times diluted hydrolysate with final concentrations glucose 12.67 g L^−1^ and xylose 6.90 g L^−1^

b medium containing three times diluted hydrolysate with final concentrations glucose 21.12 g L^−1^ and xylose 11.49 g L^−1^

## Discussion

When considering the importance of chemical feedstock, the production of high-value substances from renewable sources is becoming increasingly appealing. Lignocellulosic hydrolysates are attractive substrates in microbial fermentation processes for the production of commercially relevant compounds (Baptista et al. 2021). Previous reports have demonstrated riboflavin production from lignocellulosic hydrolysates by bacteria. Using corn cob hydrolysate as a carbon source, growth and riboflavin biosynthesis were optimized by the thermophilic strain *Geobacillus thermoglucosidasius* (Wang et al. 2022). The overexpression of the mannose-6-phosphate isomerase gene *manA*, xylose isomerase gene *xylA*, and xylulokinase gene *xylB* to enhance mannose and xylose consumption in *Corynebacterium glutamicum* resulted in a 56% increase in riboflavin productivity from sugars derived from lignocellulose breakdown (Pérez-García et al. 2021). However, the riboflavin production by the mentioned microorganisms reached only 121 mg L^−1^ and 27 mg L^−1^, which are too low for considering them as viable industrial producers of vitamin B2 at this stage.

So far, no microorganism that could efficiently produce riboflavin in lignocellulosic hydrolysates has been reported. To address this gap, we tested the ability of the *C. famata* riboflavin-overproducing strain BRPI, which was constructed in our previous work (Dmytruk et al. 2020), to grow on the main sugars found in lignocellulosic hydrolysates and to produce riboflavin. Both, hexoses (glucose, fructose, mannose, galactose) and pentoses (L-arabinose, xylose) supported growth of BRPI (Table 1). Even more intriguing was the result that BRPI synthesized riboflavin on all tested carbon sources (Table 1). We have demonstrated, for the first time, that *C. famata* is capable of overproducing riboflavin on L-arabinose and xylose. It has been shown that L-arabinose is a good carbon source for vitamin B_2_ production by recombinant *Bacillus subtilis* (Oraei et al. 2018). BRPI was also able to grow and produce riboflavin in synthetic medium with a glucose/xylose mixture and in medium with diluted sugarcane straw hydrolysate (Fig. 2, 3, 5). The overexpression of the *XYL1* gene, encoding xylose reductase, resulted in a 10–15% increase in riboflavin production in medium with diluted hydrolysate. However, additional overexpression of the *XYL2* gene, which encodes xylitol dehydrogenase, did not lead to a further increase in riboflavin synthesis. The maximally achieved riboflavin titer of around 1.5 grams per liter show promise for future research and practical applications.

This work represents the first communication, to the best of our knowledge, on the overproduction of riboflavin using lignocellulosic hydrolysate. Additional increases in riboflavin production could be attained through the two strategies involving strain construction: (i) enhancing riboflavin synthesis by activating pathways responsible for riboflavin precursors (GTP, ribulose-5-phosphate) synthesis, and (ii) further enhancing xylose utilization by overexpressing xylulokinase. It was observed that undiluted hydrolysate did not support the growth of *C. famata* strains, likely due to the inhibitory effects of furfural, HMF and acetic acid. This finding suggests the need for further work in selecting strains that are insensitive to these inhibitors. For instance, adaptive laboratory evolution could be considered as a method to achieve this goal (Ujor and Okonkwo 2022). Our results indicate that less diluted hydrolysate supports faster growth and higher riboflavin production (Fig. 5). Bearing this in mind, optimizing the fed-batch mode in bioreactors with hydrolysate as the carbon source could lead to increased riboflavin production.

Finally, it is worth emphasizing that *C. famata* is an ideal candidate for riboflavin production from lignocellulosic hydrolysates due to its robust growth and high riboflavin yield. Notably, it can efficiently utilize not only glucose and xylose but also other major sugars present in hydrolysates. These unique attributes, uncommon among native microorganism strains, offer promising opportunities for the development of industrially viable riboflavin producers using lignocellulose, pectin, beet pulp, and other relevant residues.

## Conclusions

The engineered strains of the yeast *C. famata* overproduced riboflavin on lignocellulosic hydrolysate. Riboflavin accumulation increased in the recombinant strains overexpressing the *XYL1* and *XYL2* genes, which code for xylose reductase and xylitol dehydrogenase, respectively. The highest riboflavin accumulation during bioreactor cultivation by BRPI/XYL1, using bagasse hydrolysate as the carbon source, reached 1.5 g L^−1^.

## Supplementary Material

foae020_Supplemental_Files
