# Supplementary material for: Riboflavin overproduction on lignocellulose hydrolysate by the engineered yeast Candida famata
Source: FEMS Yeast Res. 2024 Jul 15;24:foae020. doi: 10.1093/femsyr/foae020 (PMC11283204; doi:10.1093/femsyr/foae020)
Supplement: foae020_Supplemental_Files [file foae020_supplemental_files.zip › Supplementary materials 04.07.24.docx]

**Supplementary Table S1.** The composition of sugarcane straw hydrolysate prepared using liquid hot water pretreatment before enzymatic hydrolysis, following the method described by Zhuang et al., 2016; Jimenez-Gutierrez et al., 2021 and supplied by a GranBio Investimentos S.A. (Brazil).

| **Compounds** | **Concentrations (g L^-1^)** | | |
| --- | --- | --- | --- |
|  | **Dilution (times)** | | |
|  | **One** | **Three** | **Five** |
| Glucose | 63.35 | 21.12 | 12.67 |
| Xylose | 34.48 | 11.49 | 6.90 |
| Galactose | 1.20 | 0.40 | 0.24 |
| L-Arabinose | 3.43 | 1.14 | 0.69 |
| Mannose | 1.22 | 0.41 | 0.24 |
| Acetic Acid | 5.22 | 1.74 | 1.04 |
| Formic Acid | 0.93 | 0.31 | 0.19 |
| Furfural | 0.62 | 0.21 | 0.12 |
| Hydroxymethylfurfural | 0.43 | 0.14 | 0.09 |
