# Supplementary figures and images for: Riboflavin overproduction on lignocellulose hydrolysate by the engineered yeast Candida famata
Source: FEMS Yeast Res. 2024 Jul 15;24:foae020. doi: 10.1093/femsyr/foae020 (PMC11283204; doi:10.1093/femsyr/foae020)

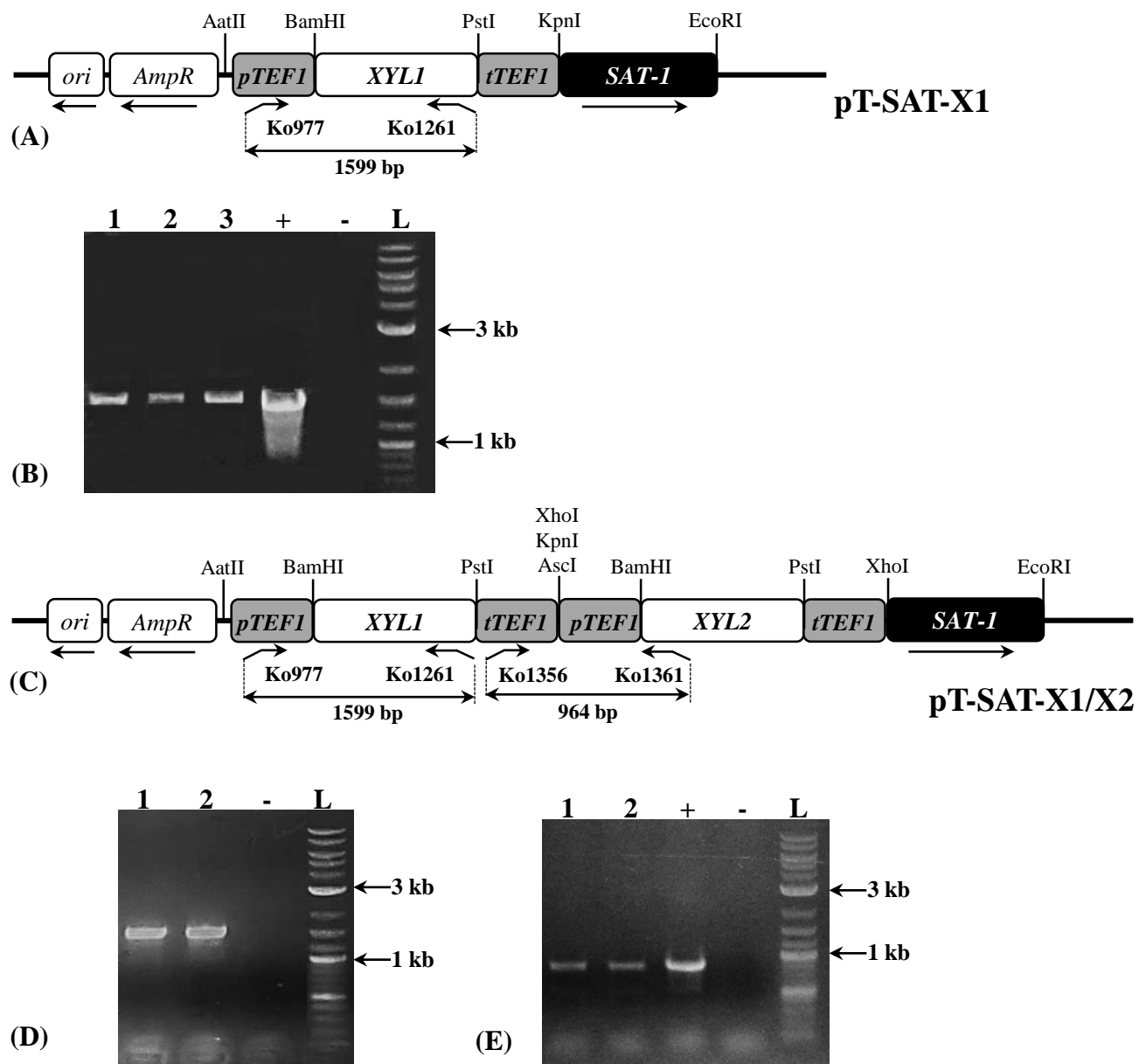

Figure 1.

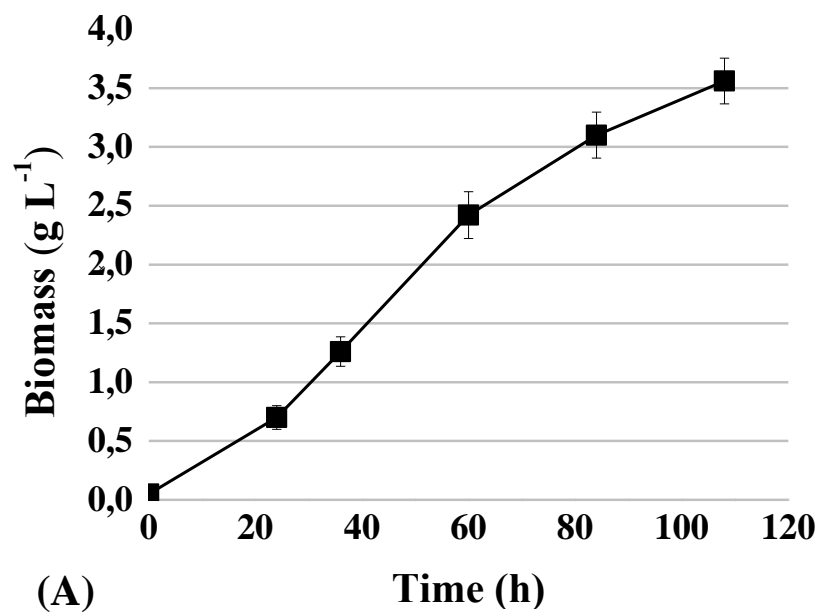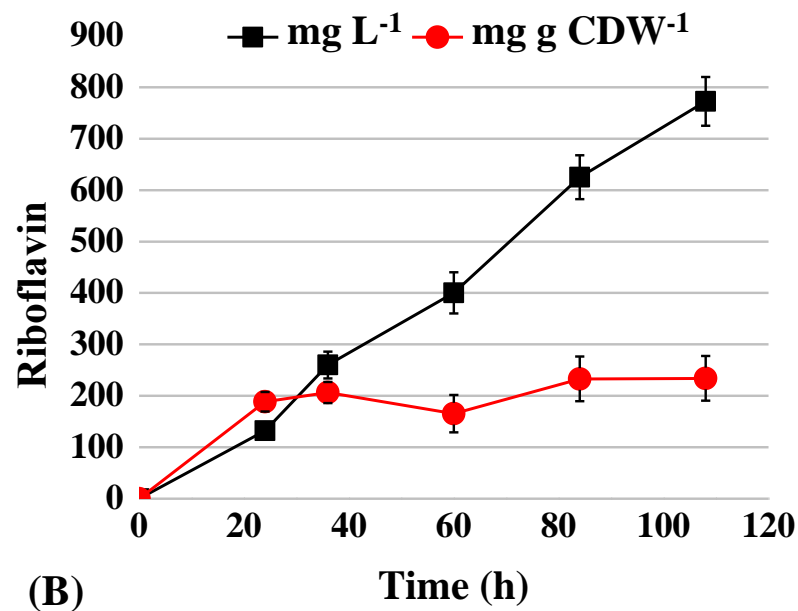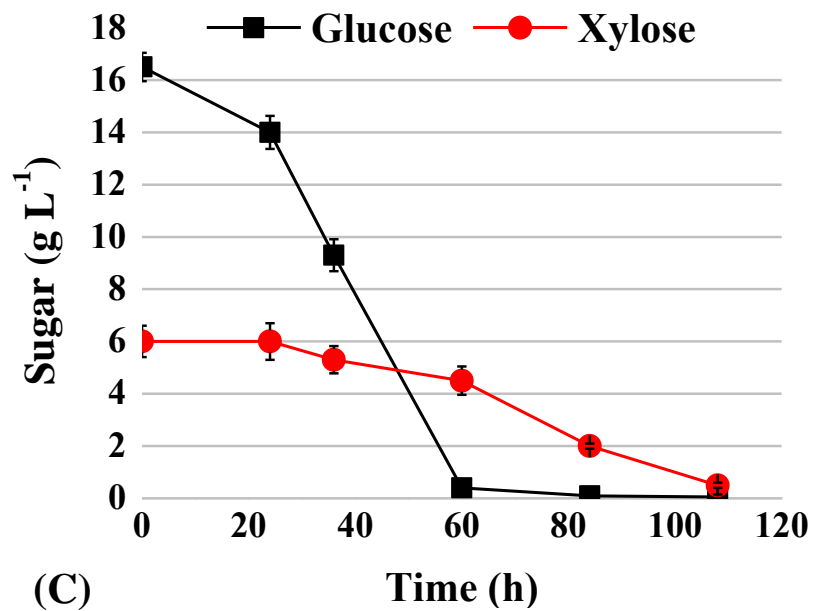

Figure 2.

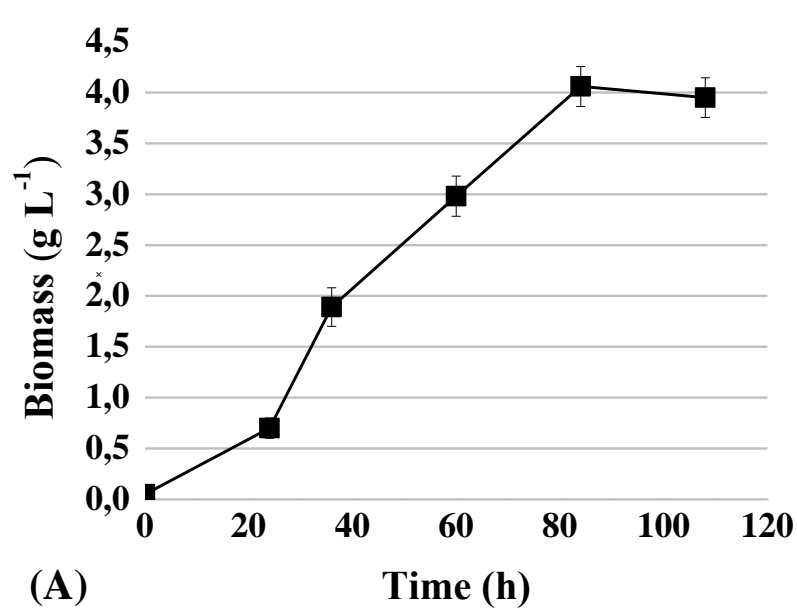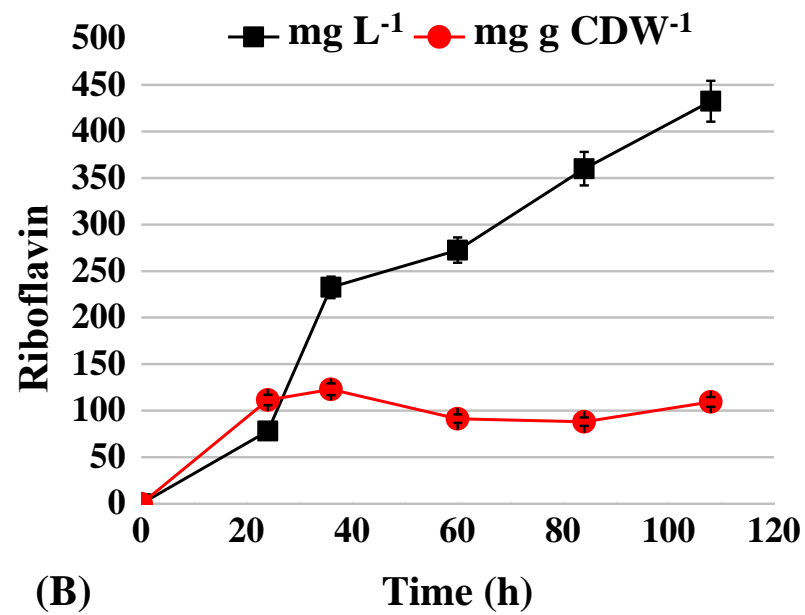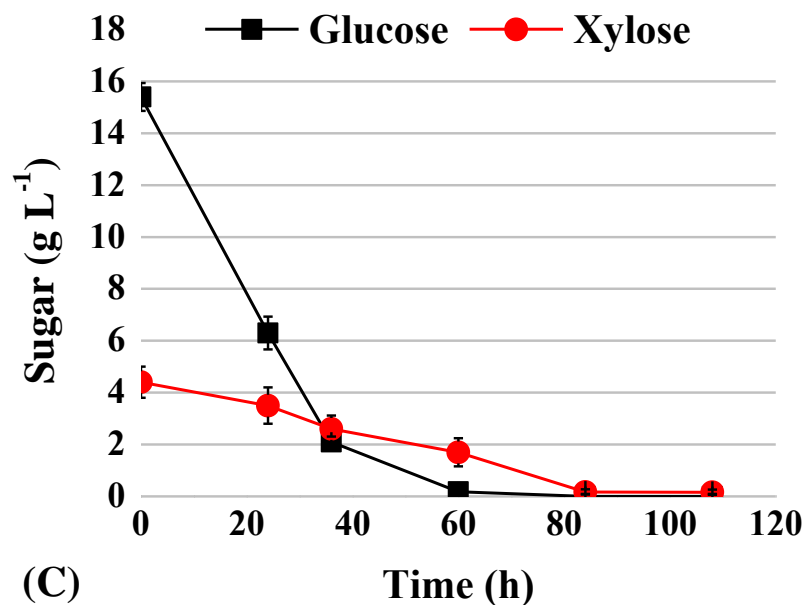

Figure 3.

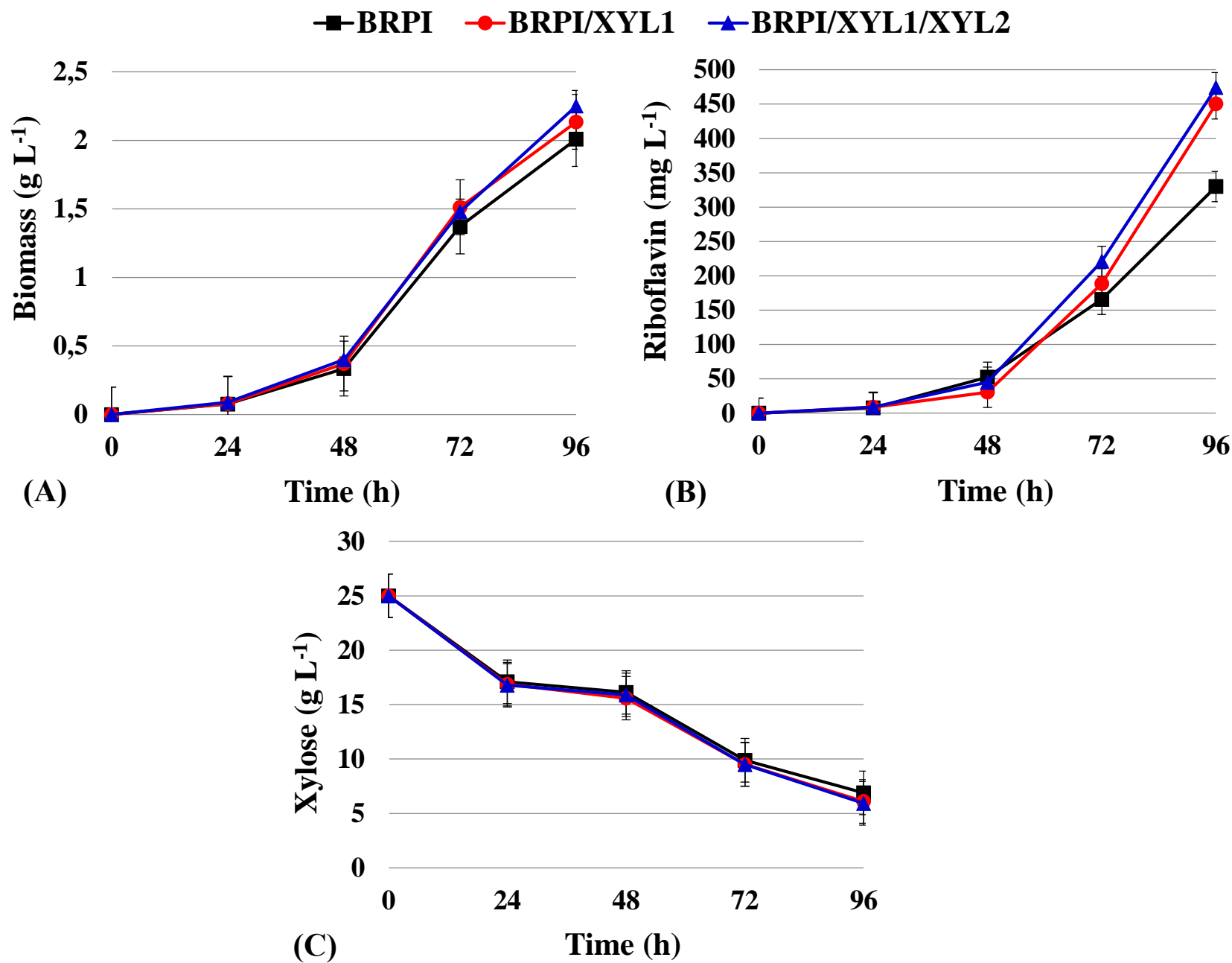

Figure 4.

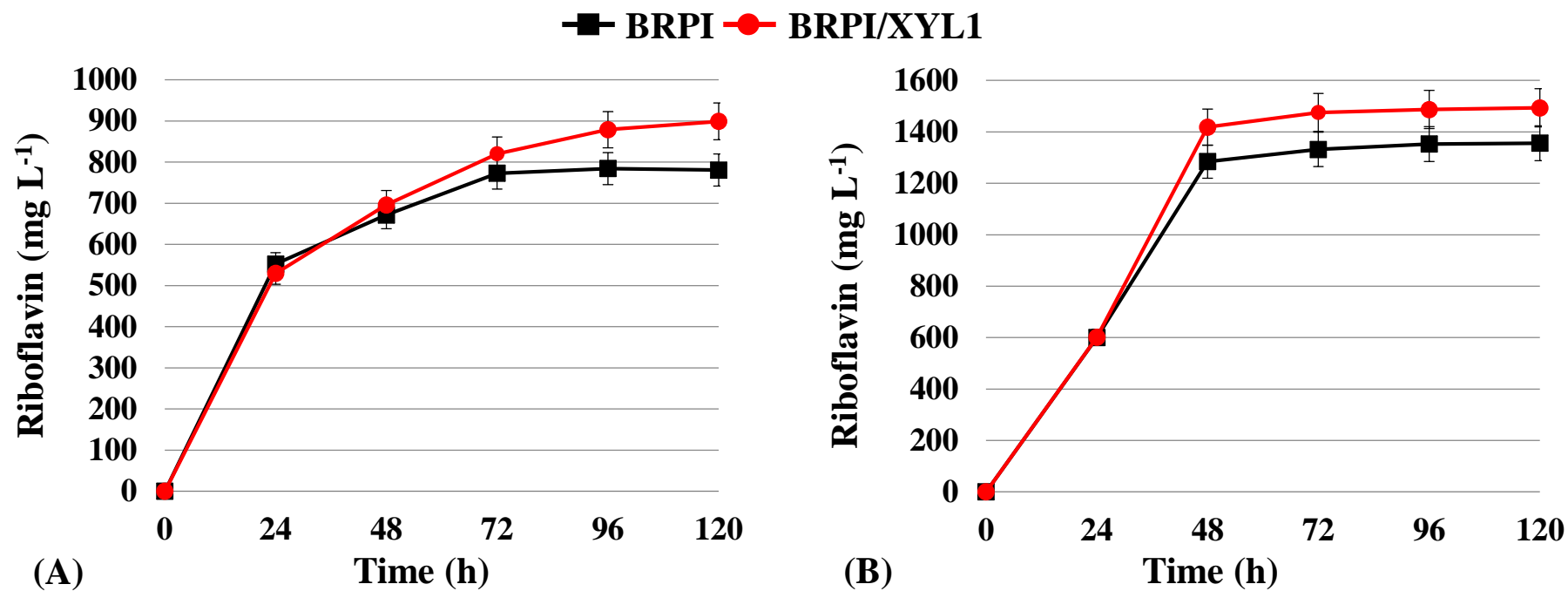

Figure 5.

Supplement: foae020_Supplemental_Files [file foae020_supplemental_files.zip › Fig_final.pdf]
